# A Network-Based Target Overlap Score for Characterizing Drug Combinations: High Correlation with Cancer Clinical Trial Results

**DOI:** 10.1371/journal.pone.0129267

**Published:** 2015-06-05

**Authors:** Balázs Ligeti, Zsófia Pénzváltó, Roberto Vera, Balázs Győrffy, Sándor Pongor

**Affiliations:** 1 Faculty of Information Technology, Pázmány Péter Catholic University, Budapest, Hungary; 2 MTA TTK Lendület Cancer Biomarker Research Group, Budapest, Hungary; 3 Protein Structure and Bioinformatics Group, International Centre for Genetic Engineering and Biotechnology, Trieste, Italy; 4 2nd Department of Pediatrics, Semmelweis University, Budapest, Hungary; 5 MTA-SE Pediatrics and Nephrology Research Group, Budapest, Hungary; Bioinformatics Institute, SINGAPORE

## Abstract

Drug combinations are highly efficient in systemic treatment of complex multigene diseases such as cancer, diabetes, arthritis and hypertension. Most currently used combinations were found in empirical ways, which limits the speed of discovery for new and more effective combinations. Therefore, there is a substantial need for efficient and fast computational methods. Here, we present a principle that is based on the assumption that perturbations generated by multiple pharmaceutical agents propagate through an interaction network and can cause unexpected amplification at targets not immediately affected by the original drugs. In order to capture this phenomenon, we introduce a novel Target Overlap Score (TOS) that is defined for two pharmaceutical agents as the number of jointly perturbed targets divided by the number of all targets potentially affected by the two agents. We show that this measure is correlated with the known effects of beneficial and deleterious drug combinations taken from the DCDB, TTD and Drugs.com databases. We demonstrate the utility of TOS by correlating the score to the outcome of recent clinical trials evaluating trastuzumab, an effective anticancer agent utilized in combination with anthracycline- and taxane- based systemic chemotherapy in HER2-receptor (erb-b2 receptor tyrosine kinase 2) positive breast cancer.

## Introduction

In the past few decades the number of novel marketed drugs has fallen much below the expectations despite the growing resources invested in this area [1–3]. Many biological pathways have rich regulatory loops which can be utilized to compensate various perturbations. In cancer therapy, drugs acting on the HER2 (erb-b2 receptor tyrosine kinase 2) and EGFR (epidermal growth factor receptor) pathways have shown this type of drug evasion effects. Multitarget drugs or drug combinations have been proposed as a general strategy to circumvent this phenomenon [4, 5] one of the reasons being that combinations often have less toxicity and higher therapeutic success [6]. The number of approved drug combinations is on the increase, even though most of them were established by experience and intuition [7, 8].

About one-fourth of breast cancer patients express HER2 (human epidermal growth factor receptor-2), a transmembrane receptor tyrosine kinase of the epidermal growth factor receptor (EGFR) family. In HER2 positive patients, administration of trastuzumab, an anti-HER2 therapy improved the progression free survival (PFS) and the overall survival (OS) [9]. It also enhanced survival as adjuvant therapy combined with chemotherapy [10] or as monotherapy after chemotherapy [11]. Since 2006, trastuzumab is also approved for use in adjuvant settings in HER2 positive early breast cancer. Anti-HER2 therapy is highly successful: although high HER2 expression was previously associated with worse survival, today HER2 positive patients have better prognosis as compared to women with HER2 negative disease [12].

According to current NCCN guidelines (www.nccn.org), trastuzumab is given in combination with adjuvant chemotherapy only. Preferred regimes for chemotherapy with trastuzumab include Adriamycin, Cyclophosphamide, Paclitaxel, Docetaxel and Carboplatin. Numerous other agents are also included in protocols used for breast cancer patients including Methothrexate, Epirubicine, Fluorouracil and protocols containing combinations of these (FAC, CAF, CMF, EC, FEC, TAC, etc.). Thus, the combination of various agents into multi-agent protocols represents the backbone of the state of the art in systemic treatment for HER2 positive breast cancer. However, finding the most efficient combinations of these is not an easy task given the complexity of the underlying biological system.

Several experimental methods, even high throughput methods [13], have been developed for measuring efficiency of drug combinations, such as Bliss independence or Loewe additivity [14–16]. Wong et al. used a stochastic search algorithm [17] while Calzoari and associates employed sequential decoding algorithms for finding the best combinations [18]. Yang et al. used differential equations to find a perturbation pattern that can revert the system from disease state to a normal state [19]. Jin and associates employed a Petri net based model to microarray data in order to predict synergism of drug pairs [20]. A common feature of these computational methods is that they require a large number of experiments or deep knowledge of the kinetic parameters of the pathways even if the search space is small.

Other studies used various combinations of data mining methods to integrate pharmacological and network data [21, 22]. Li and coworkers used the concept of network centrality and disease similarity to prioritize drug combinations [23]. Wu and associates used the microarray profile of the individual drugs for the predictions [21], and others used the concept of synthetic lethality and the available gene interaction data [24, 25]. Despite the countless attempts, there are still many challenges and open practical questions. In particular, finding suitable data representations and similarity measures is not a trivial problem because of the heterogeneity of information sources. Currently there are published data on a large number of drug combinations (six hundred in the DCDB and TTD databases as of March 2013), that refer to a variety of diseases and therapeutic targets. It is an open question whether or not the correlations and tendencies extracted from such heterogeneous datasets can be successfully applied to a specific problem, such as that of trastuzumab.

Here we present a novel principle that is based on the assumption that perturbations generated by the pharmacological agents propagate through an interaction network to other targets that constitute what we call a propagation neighborhood. Overlaps of multiple propagation neighborhoods can then cause unexpected synergies at target genes that are not in the immediate vicinity of the original targets of the individual agents. We introduce a novel Target Overlap Score (TOS) that is based on the overlap of the propagation neighborhoods of the target proteins. We show that TOS is correlated with the known efficiency of beneficial and deleterious effects of drug combinations reported in the DCDB, TTD and Drugs.com databases. We also show that there is a correlation between TOS and the outcome of recent clinical trials where trastuzumab was used in combination with anthracycline- and taxane- based systemic chemotherapy in HER2-receptor positive breast cancer.

## Results

### 2.1. TOS: A network-based Target Overlap Score for drug combinations

**Fig 1:**
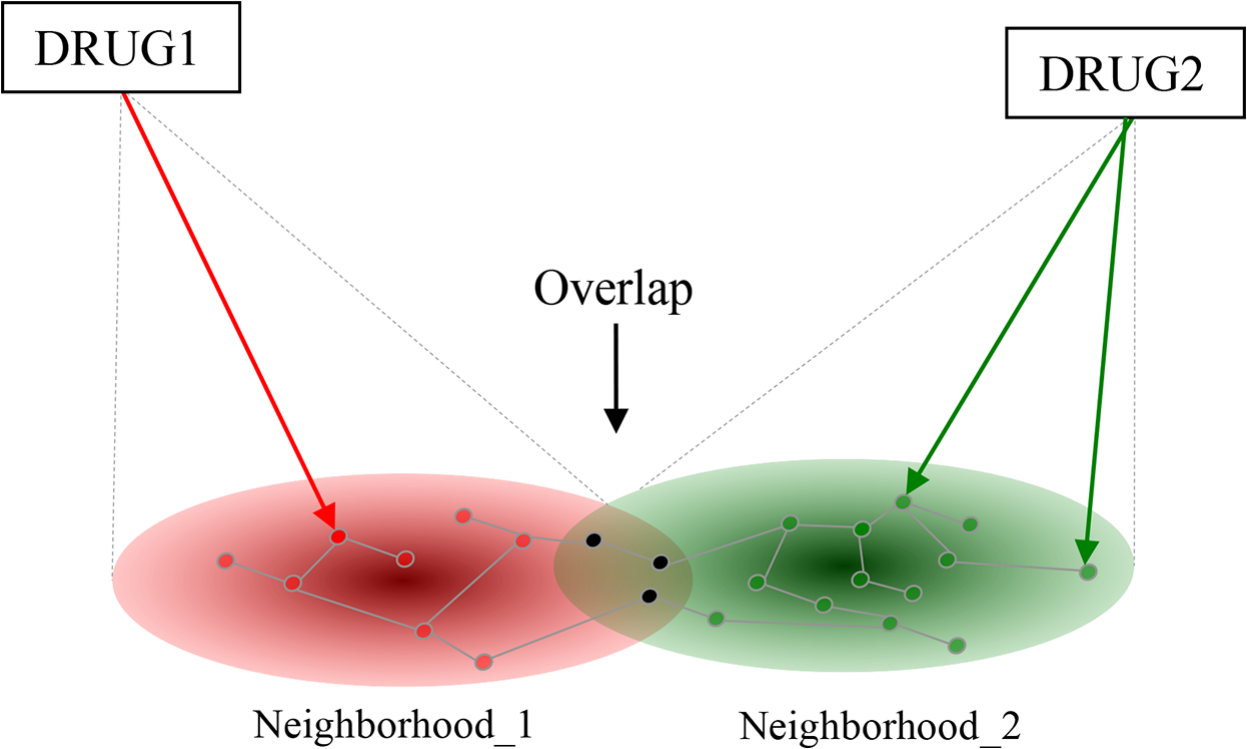
The network-interaction hypothesis. The effects of two drugs (Drug1, Drug2) reach their imminent targets first (arrows) and the effects will then propagate to their network neighborhoods (subnetworks) indicated in red and green, respectively. Targets in the overlap are affected by both drugs, and we suppose that drugs affecting a number of common targets will influence the effects of each other. The overlap is quantified as the proportion of jointly affected targets within all affected targets (in set theory terms: intercept divided by union).

Drug molecules reach their therapeutic effects by acting on specific targets in the organism and activating or inhibiting the functions of their targets. Drug effects naturally do not end here, since drug targets are members of large interaction networks through which the perturbation can propagate. For instance, by inhibiting the action of a single molecule such as BRAF (B-Raf proto-oncogene, serine/threonine kinase), the entire RAF/MEK/ERK (Raf-1 proto-oncogene, serine/threonine kinase, mitogen-activated protein kinase kinase 1, mitogen-activated protein kinase 1) pathway will be tuned down, and as a consequence, collateral pathways including PI3K (phosphatidylinositol-4,5-bisphosphate 3-kinase) and RALA (v-ral simian leukemia viral oncogene homolog A (ras related)) will also be affected. In other words, a drug acting on a single target will concomitantly perturb a group of linked targets that we term here as network neighborhood **(** Fig 1). We hypothesize that two (or more) drugs can have an unexpected combined effect if their perturbation neighborhoods overlap. In order to capture this property, we define a Target Overlap Score (TOS) for two drugs as the number of jointly affected targets divided by the number of all affected targets. This simple definition has a few plausible consequences: i) TOS has a value between zero and 1.0, higher values indicating stronger joint effects. ii) As a mathematical consequence, a drug will give TOS = 1.0 with itself. We note that even though a combination of two identical drugs does not occur in the clinical practice it can cause a statistical bias in the comparisons so they have to be removed from the datasets used in the statistical comparison (see Methods for details). iii) The concept of TOS can be generalized to more than two interacting drugs. Naturally, we have to decide in advance if, at one extreme, we want to consider genes perturbed by more than one agent in a drug combination only, or, at the other extreme, we consider just those genes that are perturbed by all of them. Here we used the former definition (for a detailed description see Data and Methods). iv) The concept of TOS does not include any supposition about the beneficial or detrimental nature of combined drug effect. This is an important point since “drug interaction” in pharmacology denote negative, detrimental effect while the term “drug combinations” usually refer to beneficial, i.e. therapeutically useful combined effects. In principle TOS can be correlated with both as we in fact show in the next chapter. v) Finally, the definition of TOS is different from several other concepts related to traditional measures of drug interactions (antagonism, agonism etc) that mostly refer to effects of drugs on the activity of one target such as a receptor. In contrast, TOS depends on the number of targets, and does not at present consider the magnitude (nor the positive or negative nature) of the effect.

### 2.2. TOS is correlated with the strength of both beneficial and deleterious drug combinations

**Fig 2:**
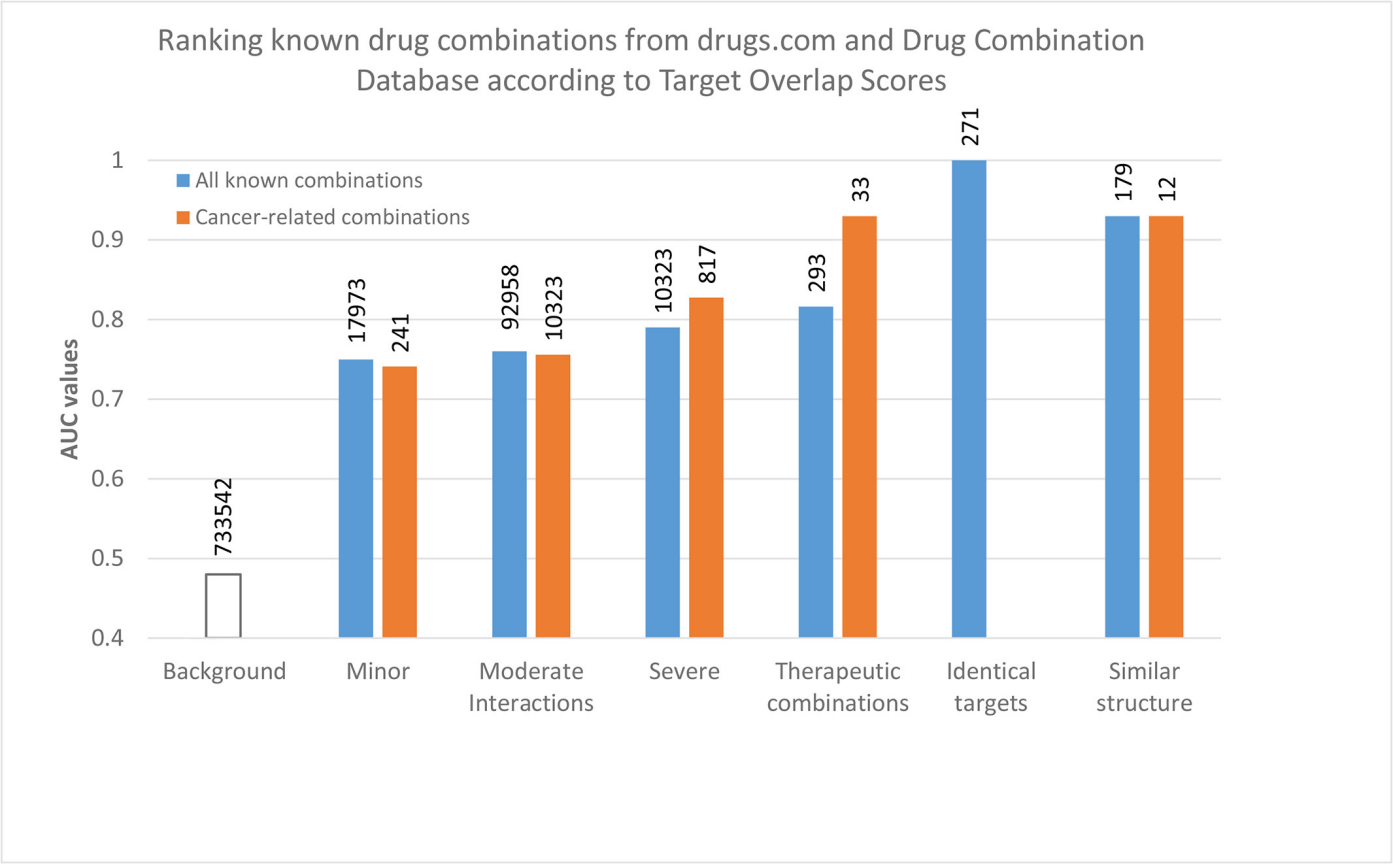
Ranking performance of the TOS score on known drug interactions and therapeutic combinations. The ranking performance was measured via ROC analysis as described in Data and Methods. The standard deviation of AUC values (not shown) are between 0.0001 and 0.006 for the different datasets. Note that the tendencies of drug combination groups are the same between cancer-related and not cancer-related drugs. Also, combinations of drugs with identical targets or with similar chemical structures give high TOS scores. These combinations were left out from the statistics of the other groups so they do not influence the AUC values of the other groups.

For the evaluation we chose a simple ranking test, i.e. we compared the TOS value calculated for known drug pairs with the TOS or randomly chosen drug pairs and calculated an AUC value for the ranking using ROC analysis [26] as described in methods (section 4.5). It is noted that strong interactions are expected to give AUC values close to 1.0 while AUC values for randomly selected pairs are expected to be around AUC ~ 0.5. In the present study we used the STRING/STITCH interaction network and the first question we asked was whether or not the evaluation system fulfils these fundamental criteria, For this purpose we used the database of FDA-approved drugs [27] and generated all possible binary combinations. Trivial interactions (drugs acting on the same target and drug pairs with identical or nearly identical chemical structures) as well as drug pairs known to have positive or negative effects were omitted from the analysis which left 733542 pairs. This set was evaluated as described in methods (Section 4.5). This evaluation gave an AUC value of 0.48 (Fig 2, left) which is very close to the random value of 0.5 This finding thus shows that, given the TOS algorithm applied to the STRING/STICTH network, the randomly chosen FDA-approved drug pairs indeed behave as random. We have to mention that the randomly selected drug pairs may have contained cases in which the interaction has not been discovered yet. A related question is that of drugs having identical targets. These should by definition give a TOS value of 1.00, and we found 271 such drug pairs. Also, drugs having close to identical chemical structures are likely to affect similar targets. We found 179 such drug pairs but only 8 of these were common with the previous subset. The comparison shows that both subsets give high TOS values which will statistically bias the comparison if included either in the positive or in the negative dataset of non-interacting drugs. So, for the statistical evaluation described below we left out these drug pairs from both datasets.

Next, we wanted to test whether or not TOS can help to identify the drug pairs that are empirically known to have a beneficial or detrimental effect. In pharmacology, two drugs are called "interacting" if their joint administration has a detrimental effect [28]. Drug pairs listed at http://drugs.com are classified into three groups according to the severity of the negative effects, such as major, moderate and minor. In the selection we considered only cancer related drug pairs i.e. those in which one of the agents was or was proposed to be used in treating cancer which resulted in 10323 strongly, 92958 moderately and 17193 weakly interacting drugs from the database, denoted as sets A, B and C, respectively (Table 1). The results show that the interacting drug pairs show remarkably higher AUC values than the randomly selected drug pairs, moreover these values qualitatively follow the strength of the interaction (Fig 2). Namely, strongly interacting drug pairs show substantially higher AUC values than the moderately interacting ones etc.

**Table 1:** Datasets.

	Dataset	Original size	Size after filtering^1^	Data source
**I. Datasets of all drugs**				
Detrimental drug interactions^2^				
Severe	A	21831	10323	Drugs.com
Moderate	B	112976	92958	Drugs.com
Minor	C	13143	17973	Drugs.com
Beneficial drug interactions^3^ ^,^ ^4^	D	429	293	DCDB, TTD
**II. Cancer-related datasets**				
Detrimental drug interactions^2^				
Severe	E	1053	817	Drugs.com
Moderate	F	6857	5700	Drugs.com
Minor	G	273	241	Drugs.com
Beneficial drug interactions^3^ ^,^ ^5^	H	55	33	DCDB, TTD
**III. Negative datasets used in ROC analysis**				
All FDA-approved drugs^6^	I	848253	733542	Drugbank
Random drugs^7^	J	427350	426425	-

^1^We filtered the available drug pairs by leaving out the drug combinations where the components have exactly the same targets, or the components were structurally similar, as described in Methods. The drugs with no available targets were also discarded

^2^Taken from Drugs.com (November 11, 2013) as described in the methods

^3^Taken from the Drug Combination Database (March 8, 2012) and the Therapeutic Target Database (July 23, 2012) as described in the methods

^4^All approved drug combinations were included

^5^All approved drug combinations that are used in cancer treatment.

^6^We made all possible binary combinations of FDA-approved drugs (taken from DrugBank, 12^th^ September of 2012), and then leaved out all pairs that were listed as beneficial or detrimental combinations.

^7^We constructed random drugs corresponding to the number of targets of all individual drugs. We generated 25 random drugs for each target count (37). From this pool we made the all possible binary combinations. In each case, we randomly selected a negative set of the size which was 5 times greater than the positive dataset [51].

We also tested drug pairs that are known to have a beneficial effect when administered together. In pharmacology, the term “drug combinations” refers to drugs that are administered together because they have an empirically known beneficial therapeutic effect. Such therapeutically useful drug combinations are included in the Drug Combination Database (DCDB) [29] as well as in the Therapeutic Target Database TTD [30], along with the specific mechanism of their interaction. Using the same selection criteria we 293 combinations (dataset D, Table 1). The results in Fig 2, right show that therapeutic drug combinations yield AUC values substantially different from the random combinations.

Next we carried out the same comparisons for cancer related drugs. In this case the datasets were naturally smaller, we found 817 strongly, 5700 moderately and 241 weakly interacting drugs from the database, and denoted as sets E, F and G, respectively (Table 1). The set of beneficial combinations included 33 combinations specifically suggested for cancer (dataset H, Table 1). The results presented in Fig 2B show the same general tendencies as seen in the case of all drug combinations (Fig 2A). Namely, i) the known interactions are substantially different from the combinations of non-interacting drugs; ii) the AUC values of minor, moderate and strong, detrimental interactions follow the correct order i.e. the stronger the interactions the higher the AUC values; and iii) the values of beneficial, therapeutic combinations is also substantially different from the average and the AUC value of 0.91 in cancer related combinations can be considered especially convincing. iv) In both panels of Fig 2, the beneficial interactions show higher AUC values. We have no ready explanation for this phenomenon, however we speculate that one of the reason could be that therapeutic combinations are usually optimized via careful clinical studies.

### 2.3. TOS vs. GO and ATC codes

**Fig 3:**
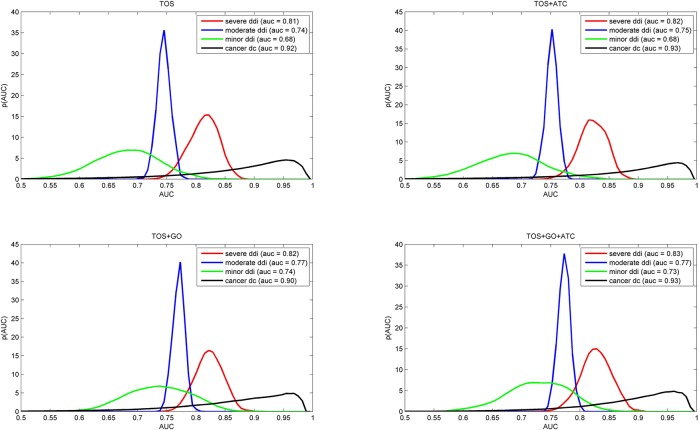
Performance of combined predictors on different training sets. The short titles TOS, TOS+ATC, TOS+GO or TOS+GO+ATC refer to the combination used. The curves represent the AUC value distribution (as a probability density function) obtained via a kernel density estimation (KDE) approach. The data were obtained by a 5 fold cross-validation procedure described in Methods (section 4.5). Note that the distributions are quite similar to the TOS values (top left) which indicates that TOS effectively captures the drug combination phenomenon.

Since TOS is conceptually different from other measures used to characterize drug interactions, one might expect that additional parameters successfully used in other studies can increase its ranking power. The most obvious way of boosting the performance of a classifier is to include more and more relevant knowledge on the drugs. Earlier studies suggest that the integration of disease similarity [23] or therapeutic information such as ATC code based similarity [22, 31, 32] as well as target similarity, such as GO annotations could be useful as well [22, 25]. In order to test these possibilities, we combined TOS with GO or ATC based similarity metrics using logistic regression [33], a standard method in machine learning studies, as described in methods. The results in Fig 3 show that inclusion of new parameters did not substantially change the picture.

The fact that the ranking power of TOS was not substantially improved when other parameters were added shows that TOS in itself captures a property that is well correlated with the empirically known interaction strength of various drug combinations.

### 2.4. TOS shows correlation with the outcome of clinical trials

In a clinical trial (also called „interventional study”), patients receive specific interventions according to a well-defined protocol [34]. In our case, trial data were collected from http://clinicaltrials.gov and consisted of studies in which combinations included trastuzumab either as an interaction partner or as a basis for comparison and only those clinical scores were used that were collected according to RECIST [35]. The list of drugs tested in clinical trials included bevacizumab, capecitabine, carboplatin, cyclophosphamide, docetaxel, doxorubicin, epirubicin, fluorouracil, gemcitabine, ixabepilone, lapatinib, oxaliplatin, paclitaxel, pertuzumab, sunitinib. All the clinical response data are listed in S1 Table.

**Fig 4:**
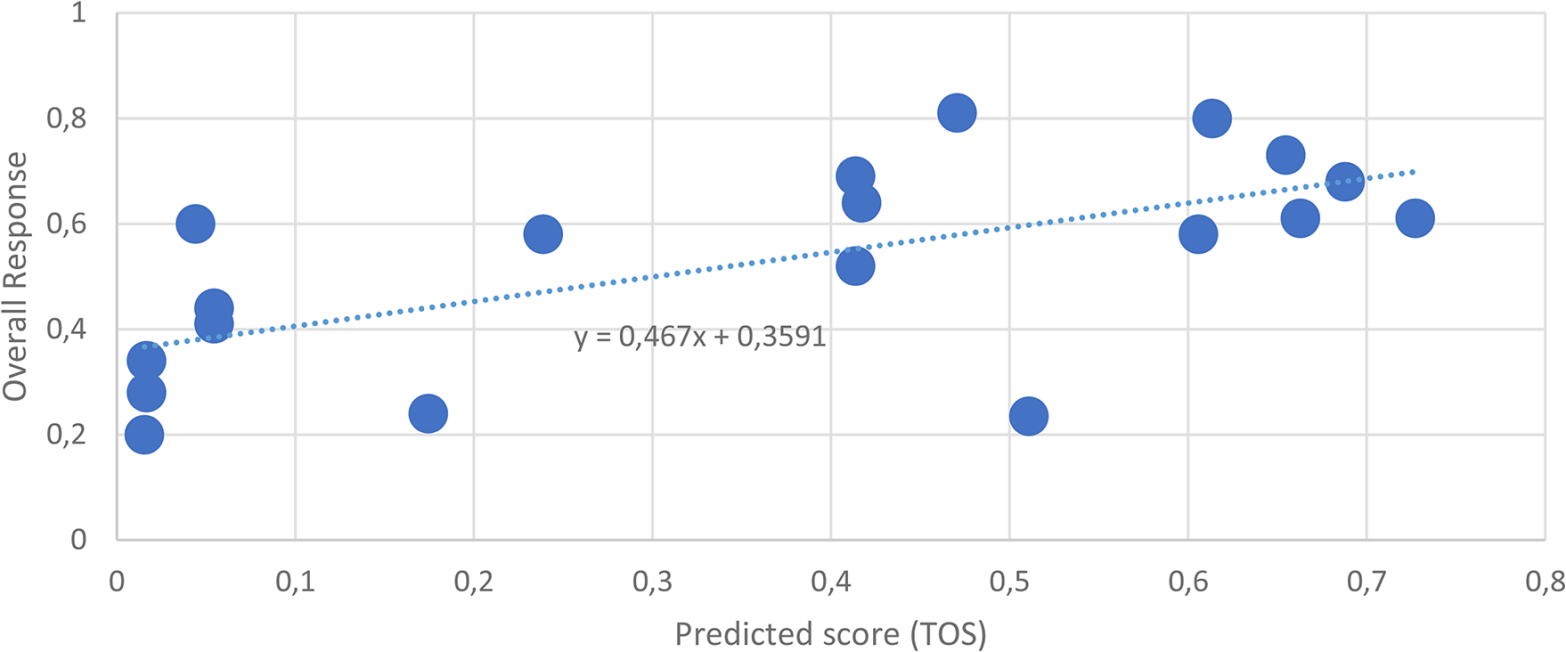
Scatter plot of TOS scores and Overall Response. The predicted scores are on the x axes, the clinical outcome, Overall Response (for the definition of outcome measures see the RECIST [35]) are on the y axes. Each data point corresponds to a multicomponent combination. The generalized TOS score of multicomponent combinations was calculated as described in Data and Methods.

First we analyze the statistical dependence between the clinical outcomes and the TOS values calculated for the drug regimens used for the treatment. Several regimens included more than two agents, such as trastuzumab and three additional drugs, A, B and C. Spearman's rank correlation coefficient was used for quantifying the statistical dependence between the TOS score and the clinical outcome measures. Table 2 shows that the TOS score shows substantial correlation with the overall response (OR) (r = 0.64; p = 0.0028)–Fig 4. Furthermore, the overall survival rate (OSR) and Confirmed Clinical Benefit (CCB) correlate well with TOS r = 0.87; p = 0.017 and r = 0.84; p = 0.0021).

**Table 2:** Spearman correlation between the clinical outcome measures and the generalized TOS scores of multicomponent combinations.

Clinical outcome^1^	r^2^	p-val.^3^
OR^4^	0.6453	0.0028
OSR^5^	0.8729	0.0175
CCB^6^	0.8440	0.0021
median PFS^7^	0.3784	0.4008

^1^All the clinical outcome measures were recorded based on the Response Evaluation Criteria in Solid Tumors (RECIST)[35]

^2^Spearman's rank correlation coefficient

^3^p-values for Spearman's rank correlation coefficient

^4^Overall Response

^5^Overall Survival Rate

^6^Confirmed Clinical Benefit

^7^Median Progression Free Survival

In conclusion, the data suggest that there is a significant correlation between the TOS scores and the outcome of clinical trials.

## Discussion

The TOS score is based on the intuitive expectation that drugs perturbing overlapping neighborhoods within a gene network will combine their effects either in the positive or in the negative sense, and that the strength of the combined effect is proportional to the ratio of jointly affected targets within all affected targets. The TOS measure will detect the overlap, but a high TOS will not tell if the variations are caused by positive or by negative synergies. Our tests showed that TOS is in a consistently good correlation with both, and that this correlation could not be substantially strengthened by including GO and ATC terms.

A special advantage of TOS is the ability to rank potential drug combinations, so in addition to the best combination it can also show how the other potential combinations perform in a relative comparison. The examples in Table 3 list cases where trastuzumab was combined with a cytotoxic drug (binary combination), or was part of a larger regimen consisting of more drugs which were studied in clinical trials. The results illustrate our message for trastuzumab: highest ranking scores were achieved by combinations containing docetaxel. In addition, the most potent single agent to be administered with trastuzumab was also docetaxel. A few agents reached low scores (cyclophosphamide, oxaliplatin, carboplatin, ixabepilone) when applied together with trastuzumab. But interestingly, some of these like cyclophosphamid and epirubicin have achieved much higher scores when applied in complex regimens which underlies the complex nature of the therapeutic response. In this context it is worth to note that we apparently cannot yet estimate whether TOS can help to predict progression free survival which is one the most important measure of clinical outcomes.

**Table 3:** TOS scores of binary and multicomponent combinations.

Trastuzumab in binary combinations^1^	TOS score^2^	Trastuzumab in multiple combinations^1^	TOS score^2^
tra+doc	0.4138	tra+lap+5fu+cyc+epi+pac	0.7272
tra+gem	0.4067	tra+5fu+cyc+epi+pac	0.6629
tra+flu	0.3888	tra+doc+sun	0.6548
tra+pac	0.3732	tra+per+doc	0.6133
tra+dox	0.2509	tra+dox+doc	0.6058
tra+epi	0.1105	tra+cap+doc	0.5108
tra+cap	0.0845	tra+bev+doc	0.4707
tra+cyc	0.0806	tra+gem+car	0.4170
tra+ixa	0.0441	tra+ixa+car	0.0544
tra+oxa	0.0155		
tra+car	0.0110		

^1^All combinations presented here were under clinical investigation as of 1st of January 2013. Components in the combinations were lapatinib (lap), fluorouracil (5fu), cyclophosphamid (cyc), epirubicin (epi), paclitaxel (pac), pertuzumab (per), docetaxel (doc), carboplatin (car), doxorubicin (dox), gemcitabine (gem), carboplatin (car), ixabepilone (ixa), oxaliplatin (oxa)

^2^The scores were computed using the generalized TOS as described in Data and Methods.

The larger the score the stronger the interaction.

Even though the correlation of TOS with drug combination data is promising, its eventual use in predictive settings has important limitations. First, TOS relies on the protein-protein (or gene-gene) interaction data available in the databases. Though such data are accumulating at a growing pace, interactions missing from the current datasets may lead to erroneous predictions. An important property of the TOS score is that it can not by itself differentiate between positive and negative effects. So a high TOS value can mean either a positive, synergistic effect or a negative, deleterious drug interaction effect. As more information becomes available on the direction, strength and type (such as inhibiton, activiation, binding, etc.) of the interactions between the drug targets, some of the above limitations will be gradually eliminated. We also mention that completely or partly identical targets will by definition lead to high TOS values. While the former are trivial, the second may be worth while to evaluate. The distinction is not built into the TOS score itself, but these cases can be identified by straightforward computations.

In addition, there is a conceptual difference between TOS and many of the other concepts of drug interactions. Namely, TOS does not limit drug-drug interactions or perturbations to identical drug targets or affected pathways. Instead, TOS captures a multitarget effect, and we think this is why the direction of the combined effect (i.e. beneficial vs. deleterious). can not be easily captured by the measure. Here a note on “beneficial” vs. “detrimental” effects is perhaps in place. Namely, many of the current, therapeutically useful drugs, including immunosuppressants or anti-cancer drugs are effective because they are toxic to a restricted population of cells. In the context of a single biochemical network, such effects would be considered as deleterious, even though in the therapeutic sense they are beneficial to the entire organism. We think it is the task of experimental studies to decide whether or not a combination with outstanding TOS score is therapeutically useful.

Summarizing, in this paper we presented the Target Overlap Score, a novel computational method for characterizing drug interactions, based on propagation neighborhoods in protein-protein interacton networks. The score is based on the hypothesis that those drugs that share a large number of perturbed proteins will have a combined effect that may be worth studying by experimental method. The ranking of the candidate combinations showed good correlation with clinical studies, so we hope this approach can contribute in the future to the design of therapeutically useful drug combinations.

## Data and Methods

### 4.1 Data sets

The protein-protein interaction data were taken from the STRING database [36] (http://string.embl.de/, retrieved on 28^th^ august of 2012). The drug related data (drug targets, synonyms, aliases, ATC codes) were taken from the Drugbank [27] via the JBioWH [37] (https://code.google.com/p/jbiowh/, retrieved on 12^th^ September of 2012), STITCH [38] (http://stitch.embl.de/, retrieved on 4^th^ September of 2012) and TTD [30] (http://bidd.nus.edu.sg/group/TTD/ttd.asp, retrieved on 23^th^ July of 2012) databases. The drug interaction data were taken from http://drugs.com/ (retrieved on 11^th^ November of 2013). The drug combination data were taken from the DCDB [29] (http://www.cls.zju.edu.cn/dcdb/, 4^th^ March of 2012), and TTD [30] (http://bidd.nus.edu.sg/group/TTD/ttd.asp, retrieved on 23^th^ July of 2012) databases.

From the STRING database the human protein-protein association and their combined confidence scores were used. From the STITCH database only those drug-protein associations were considered which had i) experimental evidence or ii) database evidence with at least 0.800 confidence, and the overall confidence was at least 0.900. Molecules such as Na^+^, Ca^2+^, ATP, etc. that had more than 45 targets were exluced from the dataset. All filtering algorithms were implented in MATLAB R2014a.

Published clinical trial data on trastuzumab were collected from the ClinicalTrials database (www.clinicaltrials.gov) using the word ‘trastuzumab’ in pairwise combination with all the 43 chemotherapeutic agents approved for breast cancer (amsacrine, azacitidine, bleomycin, cabazitaxel, capecitabine, carboplatin, carmustine, chlorambucil, cladribine, cyclophosphamide, cytarabine, dacarbazine, daunorubicin, daunorubicin (liposomal), docetaxel, doxorubicin, epirubicin, estramustine, etoposide, fludarabine, fluorouracil, gemcitabine. idarubicin, ifosfamid, irinotecan, ixabepilone, lomustine, mercaptopurine, methotrexate, mitomycin-c, mitoxantrone, nelarabine, oxaliplatin, paclitaxel, pemetrexed, pentostatin, temozolomide, teniposide, thioguanine, topotecan, vinblastine, vincristine, vinorelbine) on the 1st of January 2013. ClinicalTrials.gov is developed by the U.S National Institute of Health and contains summary information about clinical studies conducted all over the world. Only 18 agents were studied in combination with trastuzumab in 81 trials. The findings were narrowed to trials in which the effect of the combined therapy was studied (n = 43). For trials in which trastuzumab was studied in combination with more than one agent, these duplicates were included only once. Only the data recorderd according to Response Evaluation Criteria In Solid Tumors Criteria (RECIST) [35] were used. S1 Table contains the trials with clinical data. Overall clinical response (rate) (OR) was calculated from percentage of patients with complete response (CR) and partial response (PR) (OR = CR + PR) [35]. The Confirmed Clinical Benefit (CCB) was calculated from CR, PR and stable disease (CCB = CR + PR + SD) [35]. Finally the median progression free survival (PFS) and the median overall survival (OS) data were added in months.

### 4.2. The Target Overlap Score

We define a quantitative Target Overlap Score for two drugs as the ratio of jointly affected targets within all affected targets (Fig 1, above). The underlying model is that drug effects are local perturbations in a gene/protein interaction network and that perturbation can propagate along the network [39]. In order to bring this hypothesis into a testable form, we need to choose i) a method to model the perturbation of genes within a network; ii) a quantitative measure for characterizing and comparing the overlapping gene neighborhoods; and iii) networks on which to calculate the perturbations.

- Perturbations in interaction networks are often described by diffusion models (TOS) that a perturbation to spread along the edges of a network is similar to physical diffusion [40]. These models were successfully used for an analogous task, the so-called gene prioritization problem [41–44] (see Methods for details).
- We defined network neighborhood as the set of genes that are significantly perturbed by a drug. This was determined by Monte Carlo simulation, by repeating the diffusion process 10,000 times and determining the nodes (genes) whose activity changed at a chosen level significance (e.g. p<0.05) (see Methods for details). As a numerical measure for drug-drug interaction we define the Target Overlap Score (TOS) as the Jaccard coefficient (similarity measure between sets) calculated between the neighborhoods significantly affected by a pair of drugs. TOS is 1.00 for a pair of drugs affecting the same targets and 0.00 for agents that do not significantly affect any target in common. Performance of TOS (or any other combination measure) can be characterized by ranking their performance which can be determined by a standard ROC analysis/AUC calculation (see Methods) [26].
- For testing the methods we used the STRING, one of the largest available protein interaction databases [45]. The type of interactions are both physical and indirect (i.e. genes are coexpressed), furthermore the database contains a large number of predicted protein interactions.

For the actual testing we also took into consideration that additional data such as functional annotations and therapeutic information may enhance the performance of a predictive index such as TOS [22, 23, 32]. We thus integrated TOS with two other drug-similarity measures taken from the literature: a) the similarity of functions between the immediate targets (GO) [46] and b) the similarity of ATC codes (ATC) [32], respectively. For the integration of the similarity measures we trained a logistic regression model [33] that automatically weights the integrated data sources and normalizes the results. Training process needs positive and negative training sets. Here the positive training samples are known drug combinations or known drug interactions, taken from databases. However, confirmed “bad combinations” that could have been used as negative training sample are not available in such a large amount as the positive ones (only a couple of confirmed negative combinations are available in current databases). Thus we used random generated drugs as negative samples. Targets of random generated drugs are also targeted by known drugs. The reason behind this approach is that the druggable targets may be limited [2].

### 4.3. Calculation of drug interaction measures

Graph kernels can reveal important feutares of the graph structures such as network neighbourhood. We used the Regularized Laplacian Exponential Diffusion Kernel (*K*_*μ*,*α*_) (TOS) [47] for that purpose. The significance of a node being affected was estimated by Monte-Carlo simulations [48], and nodes with significance below a threshold value (typically 0.05) were considered as part of the drug target’s network neighborhood. More formally, the network is a graph *G*(*V*,*E*) where *V*, *E* are the set of nodes and edges, respectively. In this case the nodes represent genes or proteins, and the edges are the associations between them. The edges may have a weight, which can be interpreted as an association strength. Let *A* be the adjacency matrix of the graph. The element *a*_*ij*_ s the weight of the edge between node *i* and *j*, if there is no edge then it is 0.

Where *G* is a diagonal matrix, where ${\text{g}}_{i}=\sum_{j=1}^{\left|\text{V}\right|}A_{ij}$

For that purpose the Regularized Laplacian Exponential Diffusion Kernel (*K*_*μ*,*α*_) (TOS) [47]were used. The formula of that kernel is:

$$K_{\mu ,\alpha }=\sum_{k=1}^{\infty }(\frac{\alpha^{k}}{k!}{-L_{\mu })}^{k}=e^{-\alpha L_{\mu }}$$ (1)

where *L*_*μ*_ is the regularized Laplacian of the graph:

$$L_{\mu }=\mu G-A$$ (2)

The *i*th drug (*D*_*i*_) perturbation can be expressed with vector:

$$S_{DCM}({\text{D}}_{i})=K_{\mu ,\alpha }p_{0}$$ (3)

where *p*_0_

$$p_{0}=\left(\begin{array}{l} 1,\;\text{if the protein i is drug target}\\ 0,\;\text{otherwise} \end{array}\right)$$ (4)

The th element of *S*(D_*i*_) measures the disruption effect of D_*i*_ on protein *j*.

We used the parameters *μ* = 0.1 and α = 0.005 throughout this study.

Then the network neighborhood consists of the signifacntly perturbed network elements:

$$neighborhood_{drug}=\left\{v_{j}|v_{j}\in V,p\_value_{j}<0.05\right\},$$ (5)

The drug interaction measure (TOS) was then calculated as the overlap of two neighborhoods. The overlap was described by the Jacquard coefficient of the two affected sets (see Fig 1), defined as the intercept of two sets divided by the union of the sets.

$$TOS\left(drug_{i},drug_{j}\right)=\frac{\left|neighborhood_{drug_{i}}\cap neighborhood_{drug_{j}}\right|}{\left|neighborhood_{drug_{i}}\cup neighborhood_{drug_{j}}\right|},$$ (6)

This measure is easily generalized to handle complex drug regimens as well. In that case the overlap is calculated from the number of nodes that are significalty perturbed by at least two drugs divided by the size of the affected subnetwork.

$$TOS\left(drug_{1},drug_{2},\ldots ,drug_{M}\right)=\frac{\left|\underset{i,j=1\ldots M;i\neq j}{\cup }neighborhood_{drug_{i}}\cap neighborhood_{drug_{j}}\right|}{\left|\overset{M}{\underset{i=1}{\cup }}neighborhood_{drug_{i}}\right|},$$ (7)

This coefficient is zero if the two neighborhoods do not overlap and 1.0 if they are identical.

The GO similarity of two drug targets—zero if no GO terms are shared, 1.0 if all terms are shared—was calculated by the cosine coefficient as described in [46]. The GO similarity of more drug targets or neighborhoods was in an analogous manner i.e. the GO term vectors were calculated from more proteins. Formally, a GO vector (*g*_*i*_) was built for each drug in the dataset, where each entry of the vector represents the presence or the absence of a GO term annotated to the drug targets. The *i*th entry is 1 if the *i*th term is annotated to the target protein, 0 otherwise. Then the cosine similarities between drugs can be computed.

$$S_{GO}\left(D_{i},D_{j}\right)=1-\frac{g_{i}^{T}g_{j}}{\left\|g_{i}\right\|\left\|g_{j}\right\|}$$ (8)

The Anatomical Therapeutic Chemical Classification (ATC) System classifies drugs into groups at five levels in a hiearchical way. Thus the classification system can be seen as a simple ontology, more specifically a forest (disjoint union of trees). The roots of the individual trees are the first level characters/classes of the ATC system and the leaves are the full ATC codes (7 characters). There are 14 main groups at this level such as code A (Alimentary tract and metabolism), code B (Blood and blood forming organs), etc. One could quantify the similary beetween any two ATC codes with the Resnik measure [49], which is one of the most commonly used semantic similarity measure [31, 46]. The similarity is based on the common ancestors’s information content (IC), that quantifies the specificity and the informativity of an ATC code level. The IC of an ATC code level (c) is the negative log likelihood of the probability of the code (*p*(c)) being used. This can be estimated from the annotation frequency.

IC(c)=−logp(c) (9)

The Resnik similarity is the most informative common ancestor (MICA) of two ATC codes:

$$sim\left(\text{ATC}\_{\text{code}}_{i},\text{ATC}\_{\text{code}}_{j}\right)=\text{IC}\left(MICA\left(\text{ATC}\_{\text{code}}_{i},\text{ATC}\_{\text{code}}_{j}\right)\right)$$ (10)

Then one can compute the similarites between drugs by taking the maximum value of all possible pairwise Resnik similarities between each set of ATC codes annotated to the two drugs.

The ATC similarity is 0 if the drugs do not share any ATC codes at any level, 1.0 if they have the exactly same ATC code annotation.

**Fig 5:**
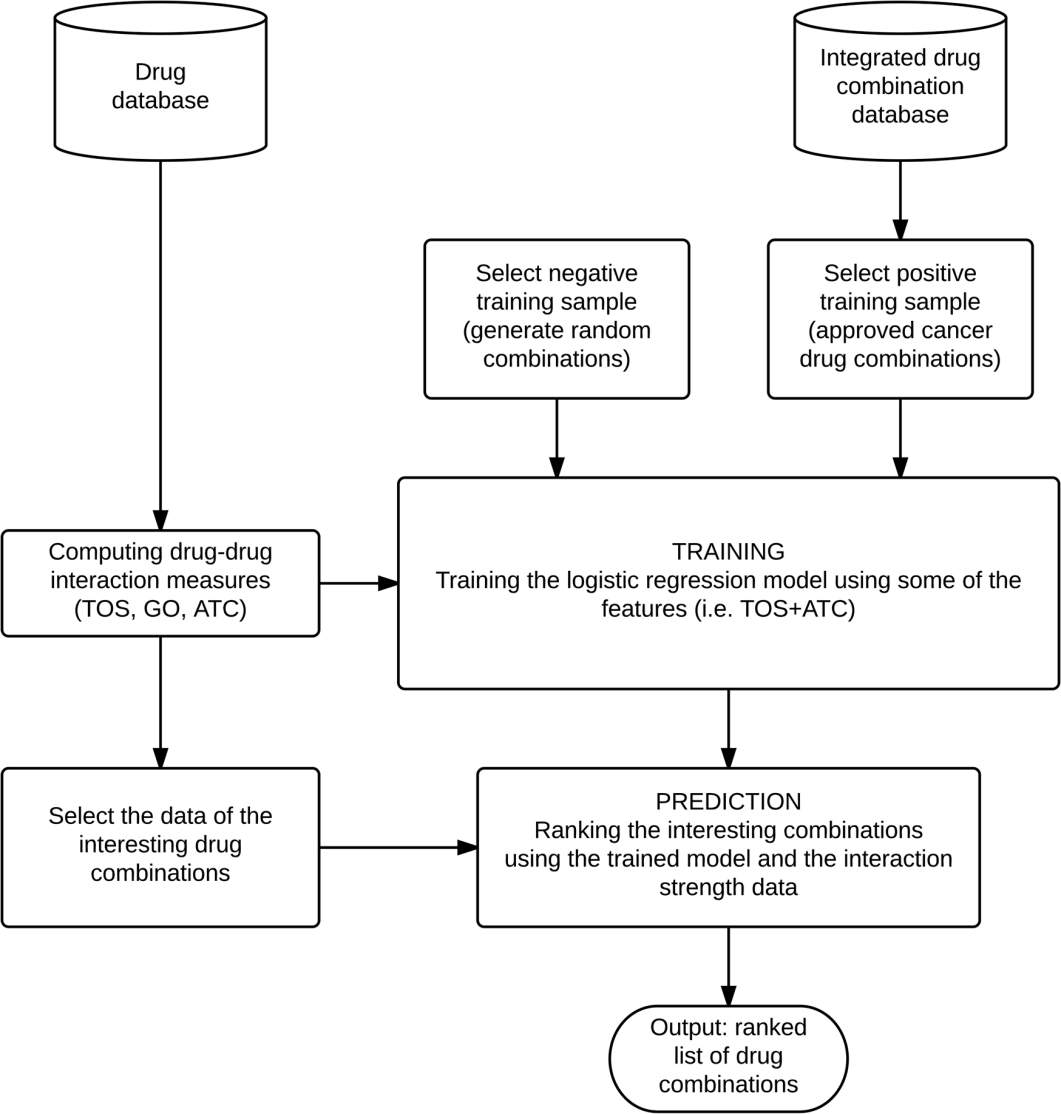
Flow chart of the training procedure. The input is a list of candidate combinations (i.e. combinations selected for clinical trials) and the set of known combinations (i.e. previously approved cancer combinations). The first step is to compute the Target Overlap Score (TOS) and the drug interaction measures (GO, ATC) for all possible drug combinations. The database consists of the random generated drugs and of the components of the candidate and the known combinations. After the selection of the training sample (both the positive—known cancer combinations—and the negative one—random combinations) a logistic regression was trained using the previously computed TOS and similarity values. In the next step the trained model is used for ranking a set of candidate combinations. The output is the ranked list of the drug combinations.

The Target Overlap Scores were then calculated for each drug pair in a database as shown in Fig 5.

### 4.4. Combining drug-drug interaction measures

Pairwise and ternary combinations of the three interaction measures (TOS, GO, ATC) were calculated by the logistic regression model [33]. Briefly, for a series *m*_1_,*m*_2_,…,*m*_*n*_ measures to be combined, the logical regression model will calculate a combined measure *M* as

$$M\left(m_{1},m_{2},\ldots ,m_{n}\;\right)=\frac{1}{1+e^{\beta_{0}+\sum \beta_{i}m_{i}}}$$ (11)

where the *β*_*i*_ regression coefficients are estimated by linear regression for which we used the *glmfit* function of MATLAB (http://www.mathworks.com/help/stats/glmfit.html). For parameter estimation, the logical regression model uses positive and negative examples. In our case, the positive examples were the known drug combinations taken from the DCDB and the TTD databases [29, 30]. The full evaluation pipeline is shown in Fig 5.

The data in Fig 3 were obtained using a 5 fold cross validation procedure for calculating the combined measures using one of the four datasets indicated in the figure, combined with a 5 fold excess number of random generated drug pairs as the negative set. The cross validation procedure was repeated 100 times with different negative sets and the estimated distribution of AUC values is shown in the figure.

### 4.5. ROC analysis

The performance of the numerical indices and index combinations was characterized by their ability to rank positive and negative examples. We chose the method of ROC analysis as it provides a robust measure of the ranking ability of parameters [26]. In our case the choice of the negative sets was problematic, since the number of experimentally validated negative, unsuccessful drug combinations is negligeable in the published databases and, opn the other hand, many of the interacting drugs are apparently multitarget pharmacons while the majority of FDA/approved drugs have one single target in the databases. We used two strategies (a and b) to construct negative samples that circumvent these problems. a) One approach was to construct binary combinations from FDA-approved drugs and leave out those combinations that are known to have beneficial or detrimental effects. b) Random drug combinations as negative datasets. A random drug is defined as a set of randomly selected target annotated with a randomly selected ATC code. The target distribution of the drug components in the negative sample was the same as in the positive set. The targets of random drugs also have to be targeted by at least one known, FDA approved drug. 427350 random combinations were generated in this way. We also defined two kinds of “trivial combinations” which were separately characterized.: i) Drug combinations having exactly the same targets in the network and ii) Combinations consisting of structurally similar components. The structural similarity was computed with the cdk module of Cinfony toolkit [50] and drug pairs with a Tanimoto coefficient is > 0.85 (computed based on the extended fingerprints) were excluded. These two subesets were then separately tested and the underlying combinations were excluded from the statistics of the other positive sets.

The ranking performance was calculated by ROC analysis [26], using an 5-fold cross-validation (CV) process [51]. We repeated the the CV process 100 times using a new set of negative samples in each round, and the average and standard deviation of (test) AUC values were reported. Ass a rule, we used random drug combinations as the negative set. We also tested the combinations of FDA approved drugs, and the tendencies of the results were found to be identical with those in Fig 2 (data not shown). Also, the differences were found significant according to the Wilcoxon test S2 and S3 Tables, however we do not attribute special importance to this fact. Namely, the difference between say moderate and minor interactions in the drugs.com database seems to be qualitative, moreover there is an unexplicable number of drug combinations that are listed both as minor and as moderate (S4 Table).

## 5. Supporting Information

S1 Table Clinical Trial Data. ^1^All the clinical outcome measures were recorded based on the Response Evaluation Criteria in Solid Tumors (RECIST) [35]; ^2^Components in the combinations: tra, lap, 5fu, cyc, epi, pac, per, doc, car, dox, gem, car, sun, ixa, oxa are trastuzumab, lapatinib, fluorouracil, cyclophosphamid, epirubicin, paclitaxel, pertuzumab, docetaxel, carboplatin, doxorubicin, gemcitabine, carboplatin, sunitinib, ixabepilone, oxaliplatin; ^3^ The phase category of the given clinical study. ^4^Confirmed Clinical Benefit; ^5^Overall Response; ^6^Pathological Complete Response; ^7^Complete Response; ^8^Partial Response; ^9^Stable Disease ^10^Progressive Disease; ^11^Overall Survival Rate (in weeks); ^12^median Progression free survival (in month);
(DOC)

S2 Table Results of two-sided Wilcoxon rank sum test for all against all comparisons of TOS scores of known drug combinations. ^1^All drug–drug interaction data taken from Drugs.com (November 11, 2013) and Drug Combination Database (March 8, 2012) as described in Methods. The TOS scores of each dataset were compared to all the other datasets and then the p-value was reported; ^2^All the known detrimental and beneficial drug interactions; ^3^All cancer related drug combinations; ^4^For detailed description of datasets see Table 1.
(DOC)

S3 Table Results of two-sided Wilcoxon rank sum test for TOS scores of known combinations. ^1^For detailed description of datasets see Table 1; ^2^The results from two-sided Wilcoxon rank sum tests comparing the TOS scores of known combinations to the scores of random combinations.
(DOC)

S4 Table Drug interactions tagged as either severe, moderate or minor. ^1^Name of the interaction partner taken from drugs; ^2^Drug labels taken from drugs.com
(XLS)
