# Supplementary material for: A Network-Based Target Overlap Score for Characterizing Drug Combinations: High Correlation with Cancer Clinical Trial Results
Source: PLoS One. 2015 Jun 5;10(6):e0129267. doi: 10.1371/journal.pone.0129267 (PMC4457853; doi:10.1371/journal.pone.0129267)
Supplement: S1 Table — 1All the clinical outcome measures were recorded based on the Response Evaluation Criteria in Solid Tumors (RECIST) [35]; 2Components in the combinations: tra, lap, 5fu, cyc, epi, pac, per, doc, car, dox, gem, car, sun, ixa, oxa are trastuzumab, lapatinib, fluorouracil, cyclophosphamid, epirubicin, paclitaxel, pertuzumab, docetaxel, carboplatin, doxorubicin, gemcitabine, carboplatin, sunitinib, ixabepilone, oxaliplatin; 3 The phase category of the given clinical study. 4Confirmed Clinical Benefit; 5Overall Response; 6Pathological Complete Response; 7Complete Response; 8Partial Response; 9Stable Disease 10Progressive Disease; 11Overall Survival Rate (in weeks); 12median Progression free survival (in month); (DOC) [file pone.0129267.s001.doc]

# Supporting Information

**TABLE S1. Clinical Trial Data**

|  | | | Clinical outcome measures^1^ | | | | | | | | |
| --- | --- | --- | --- | --- | --- | --- | --- | --- | --- | --- | --- |
| Clinical Trial ID | COMBINATION^2^ | Phase^3^ | CCB^4^ | OR^5^ | PCR^6^ | CR^7^ | PR^8^ | SD^9^ | PD^10^ | OSR^11^ | median PFS^12^ |
| NCT00524303 | lap+tra+5fu+cyc+epi+pac | II |  | 0,61 | 0,74 |  |  |  |  |  |  |
| NCT00567190 | per+tra+doc | III |  | 0,80 |  |  |  |  |  |  | 18,50 |
| NCT00524303 | tra+5fu+cyc+epi+pac | II |  | 0,61 | 0,54 |  |  |  |  |  |  |
| NCT00364611 | tra+bev+doc | II | 0,86 | 0,81 |  | 0,19 | 0,62 | 0,05 |  |  |  |
| NCT00464646 | tra+bev+doc+cyc+epi | II |  |  | 0,47 |  |  |  |  |  |  |
| NCT00127933 | tra+cap+doc | IV |  | 0,24 | 0,36 |  |  |  |  | 94,12 |  |
| NCT00567190 | tra+doc | III |  | 0,69 |  |  |  |  |  |  | 12,40 |
| NCT00490646 | tra+doc | II | 0,88 | 0,52 |  | 0,08 | 0,44 | 0,36 | 0,04 |  | 13,00 |
| NCT00232479 | tra+doc+car | II |  |  | 0,43 |  |  |  |  |  |  |
| NCT00687440 | tra+doc+dox | III | 1,00 | 0,58 |  | 0,08 | 0,50 | 0,42 |  |  |  |
| NCT00372424 | tra+doc+sun | I |  | 0,73 |  |  |  |  |  |  | 14,60 |
| NCT00191451 | tra+gem+car | II | 0,88 | 0,64 |  | 0,12 | 0,52 | 0,24 | 0,08 | 73,30 |  |
| NCT00490646 | tra+ixa | II | 0,84 | 0,60 |  | 0,12 | 0,48 | 0,24 | 0,04 |  | 11,30 |
| NCT00077376 | tra+ixa+car | II | 0,67 | 0,41 |  | 0,08 | 0,33 | 0,26 | 0,28 | 50,00 |  |
| NCT00077376 | tra+ixa+car | II | 0,69 | 0,44 |  | 0,07 | 0,37 | 0,25 | 0,27 | 48,00 |  |
| NCT00297596 | tra+oxa | II |  | 0,20 |  |  |  |  |  |  |  |
| NCT00127933 | cap+doc | IV |  | 0,24 | 0,10 |  |  |  |  | 96,72 |  |
| NCT00364611 | doc+bev | II | 0,85 | 0,58 |  | 0,06 | 0,52 | 0,27 |  |  |  |
| NCT00004888 | dox+doc | II |  |  |  |  |  |  |  |  | 11,00 |
| NCT00191451 | gem+car | II | 0,70 | 0,28 |  | 0,00 | 0,28 | 0,43 | 0,26 | 41,40 |  |
| NCT00191451 | gem+car | II | 0,62 | 0,34 |  | 0,02 | 0,32 | 0,28 | 0,36 | 20,50 |  |
| NCT00524303 | lap+5fu+cyc+epi+pac | II |  | 0,68 | 0,45 |  |  |  |  |  |  |

*^1^All the clinical outcome measures were recorded based on the Response Evaluation Criteria in Solid Tumors (RECIST) ; ^2^Components in the combinations: tra, lap, 5fu, cyc, epi, pac, per, doc, car, dox, gem, car, sun, ixa, oxa are trastuzumab, lapatinib, fluorouracil, cyclophosphamid, epirubicin, paclitaxel, pertuzumab, docetaxel, carboplatin, doxorubicin, gemcitabine, carboplatin, sunitinib, ixabepilone, oxaliplatin; ^3^ The phase category of the given clinical study. ^4^Confirmed Clinical Benefit; ^5^Overall Response; ^6^Pathological Complete Response; ^7^Complete Response; ^8^Partial Response; ^9^Stable Disease ^10^Progressive Disease; ^11^Overall Survival Rate (in weeks); ^12^median Progression free survival (in month);*
