# Supplementary material for: A Network-Based Target Overlap Score for Characterizing Drug Combinations: High Correlation with Cancer Clinical Trial Results
Source: PLoS One. 2015 Jun 5;10(6):e0129267. doi: 10.1371/journal.pone.0129267 (PMC4457853; doi:10.1371/journal.pone.0129267)
Supplement: S2 Table — 1All drug–drug interaction data taken from Drugs.com (November 11, 2013) and Drug Combination Database (March 8, 2012) as described in Methods. The TOS scores of each dataset were compared to all the other datasets and then the p-value was reported; 2All the known detrimental and beneficial drug interactions; 3All cancer related drug combinations; 4For detailed description of datasets see Table 1. (DOC) [file pone.0129267.s002.doc]

**Table S2: Results of two-sided Wilcoxon rank sum test for all against all comparisons of TOS scores of** known drug combinations.

|  | |  | ALL KNOWN DRUG INTERACTIONS AND COMBINATIONS^1,2^ | | | | CANCER RELATED DRUG INTERACTIONS AND COMBINATIONS^1,3^ | | | |
| --- | --- | --- | --- | --- | --- | --- | --- | --- | --- | --- |
|  |  |  | Detrimental drug interactions | | | Beneficial drug interactions | Detrimental drug interactions | | | Beneficial drug interactions |
|  |  |  | Severe | Moderate | Minor | - | Severe | Moderate | Minor | - |
|  |  | Dataset^4^ | A | B | C | D | E | F | G | H |
| Detrimental drug interactions | Severe | A | - | 1.06E-105 | 1.87E-87 | 0.0069 | 6.37E-13 | 2.56E-10 | 0.0024 | 1.32E-06 |
|  | Moderate | B | - | - | 7.69E-09 | 2.22E-08 | 1.03E-33 | 3.92E-08 | 0.8150 | 3.38E-08 |
|  | Minor | C | - | - | - | 3.26E-11 | 2.82E-42 | 9.75E-16 | 0.5252 | 3.82E-09 |
| Beneficial drug interactions | - | D | - | - | - | - | 0.1823 | 3.30E-05 | 3.73E-05 | 0.0003 |
| Detrimental drug interactions | Severe | E | - | - | - | - | - | 8.87E-20 | 7.03E-10 | 0.0008 |
|  | Moderate | F | - | - | - | - | - | - | 0.1349 | 8.44E-07 |
|  | Minor | G | - | - | - | - | - | - | - | 2.59E-07 |
| Beneficial drug interactions | - | H | - | - | - | - | - | - | - | - |

^1^All drug – drug interaction data taken from Drugs.com (November 11, 2013) and Drug Combination Database (March 8, 2012) as described in the Methods. The TOS scores of each dataset were compared to all the other datasets and then the p-value was reported; ^2^All the known detrimental and beneficial drug interactions; ^3^All cancer related drug combinations. ^4^For detailed description of datasets see **TABLE 1**.
