# Supplementary material for: A Network-Based Target Overlap Score for Characterizing Drug Combinations: High Correlation with Cancer Clinical Trial Results
Source: PLoS One. 2015 Jun 5;10(6):e0129267. doi: 10.1371/journal.pone.0129267 (PMC4457853; doi:10.1371/journal.pone.0129267)
Supplement: S3 Table — 1For detailed description of datasets see Table 1; 2The results from two-sided Wilcoxon rank sum tests comparing the TOS scores of known combinations to the scores of random combinations. (DOC) [file pone.0129267.s003.doc]

**Table S3: Results of two-sided Wilcoxon rank sum test for TOS scores of known combinations.**

|  | | Dataset^1^ | p-value^2^ |
| --- | --- | --- | --- |
| Detrimental drug interactions | Severe | A | 0 |
|  | Moderate | B | 0 |
|  | Minor | C | 0 |
| Beneficial drug interactions | - | D | 4.56E-66 |
| Cacner related detrimental drug interactions | Severe | E | 1.43E-190 |
|  | Moderate | F | 0 |
|  | Minor | G | 4.94E-33 |
| Cancer related beneficial drug interactions | - | H | 7.81E-15 |

^1^For detailed description of datasets see **TABLE 1.;** ^2^The results from two-sided Wilcoxon rank sum tests comparing the TOS scores of known combinations to the scores of random combinations.
